# Mitigation strategies and compliance in the COVID-19 fight; how much compliance is enough?

**DOI:** 10.1371/journal.pone.0239352

**Published:** 2021-08-09

**Authors:** Swati Mukerjee, Clifton M. Chow, Mingfei Li

**Affiliations:** 1 Department of Economics, Bentley University, Waltham, Massachusetts, United States of America; 2 Academic Technology Center, Bentley University, Waltham, Massachusetts, United States of America; 3 Department of Mathematical Science, Bentley University, Waltham, Massachusetts, United States of America; University of Zambia, ZAMBIA

## Abstract

The U.S. with only 4% of the world’s population, bears a disproportionate share of infections in the COVID-19 pandemic. To understand this puzzle, we investigate how mitigation strategies and compliance can work together (or in opposition) to reduce (or increase) the spread of COVID-19 infection. Building on the Oxford index, we create state-specific stringency indices tailored to U.S. conditions, to measure the degree of strictness of public mitigation measures. A modified time-varying SEIRD model, incorporating this Stringency Index as well as a Compliance Indicator is then estimated with daily data for a sample of 6 U.S. states: New York, New Hampshire, New Mexico, Colorado, Texas, and Arizona. We provide a simple visual policy tool to evaluate the various combinations of mitigation policies and compliance that can reduce the basic reproduction number to less than one, the acknowledged threshold in the epidemiological literature to control the pandemic. Understanding of this relationship by both the public and policy makers is key to controlling the pandemic. This tool has the potential to be used in a real-time, dynamic fashion for flexible policy options. Our methodology can be applied to other countries and has the potential to be extended to other epidemiological models as well. With this first step in attempting to quantify the factors that go into the “black box” of the transmission factor *β*, we hope that our work will stimulate further research in the dual role of mitigation policies and compliance.

## Introduction

By July 31, 2020, the COVID-19 pandemic had resulted in 31.65 million infections and 971,711 deaths globally [1] with the U.S. contributing 6.9 million cases, with 200,818 deaths. Though testing accelerated over the summer, on August 13, 2020, the U.S. had an overall positivity rate (the percentage of tests conducted that are positive for COVID-19) of 7.5% [2], well above the upper bound recommended by the WHO (World Health Organization). Before reopening is considered, the positivity rate should be 5% or below for at least 14 days [3]. Why is the U.S. then bearing such a disproportionate burden of infective cases when it has only 4.25% of the world’s population? [4].

Clues to this conundrum can only be seen by disaggregating to the state level where we encounter considerable heterogeneity. On August 16, 2020, only 17 states had met the positivity recommendations [5]. Further, the state disparities are a confusing mosaic of community mitigation strategies (with varying degrees of strictness) and diverse degrees of compliance by the public to such policies.

The objective of this study is to investigate how mitigation strategies and compliance can work together (or in opposition) to reduce (or increase) the spread of infection. To accomplish this, we build an epidemiological model that specifically takes into account not only the community mitigation strategies that slow the spread of the virus, but also compliance by the public. The importance of compliance is generally acknowledged and can be seen, for instance, in the controlling of the Ebola outbreak [6] where even a day’s delay in full compliance could double the number of infections. The model is applied to a sample of 6 U.S. states (New York, New Hampshire, New Mexico, Colorado, Texas, and Arizona) chosen for three reasons: varying success in flattening the infection curve; availability of daily data on recoveries needed for estimation; a range of positivity ratios. As of August 16 2020, the positivity ratios were 0.83 for New York, 1.34 for New Hampshire, 2.59 for New Mexico, 3.83 for Colorado, 15.32 for Texas and 10.79 for Arizona. Based on the estimation of our model, we offer some recommendations and a practical tool to aid public policy in combating the pandemic.

Apart from widespread vaccination, mitigation actions are the primary bulwark against COVID-19 and this is well established in the literature. Anderson, Heesterbeek, & Hollingsworth [7], discuss the importance of various country-wide mitigation measures. Kucharski, et al. [8] investigate the effect of isolation, contact tracing, testing and physical distancing on reducing transmission. Hellewell, et al. [9] show that in most cases if contact tracing is effective then together with isolation, three months would be sufficient to control COVID-19. Prem et al. [10] looked at physical distancing measures in Wuhan, China and concluded that these potentially could both reduce and flatten the peak of the epidemic. In fact they warned that a too early and sudden removal of these restrictions could precipitate a secondary peak. To give a historical context, Hatchett, Mecher, & Lipsitch [11] and Bootsma & Ferguson [12] inform us on public health intervention measures during the 1918 pandemic. These ranged from the individual level like washing hands, wearing face masks, and maintaining physical distance to those imposed by authorities such as restrictions on gatherings, school and workplace closings etc. Teslya, et al. [13] showed the importance of mask wearing and hand washing in conjunction with social distancing. Mask wearing and handwashing are two measures entirely within the control of the individual, whereas other measures may need group cooperation. These recommendations can, however, be ignored and that is where the importance of compliance comes in. The Oxford University Blavatnik School of Government has created an index to capture such strategies [14]. With data from more than 160 countries including the U.S., it has calculated, for each country, a Government Response Stringency Index (GRSI), scaled from 0 to 100, using 9 indicators of mitigation such as school closings, restrictions on gatherings and so on [15]. See Appendix A in S1 Appendix for the individual indicators. As of July 31, 2020, the Index for the U.S. is 69.0 and to give this some context, it is 67.13 for Canada, 68.06 for Australia and 81.94 for China. Very recently, it has also constructed Stringency Indices for each U.S. state [14].

The national GRSI has been used by researchers [16] and has also been used in a modified version applied to Brazil [17]. We build on and extend the contribution by the Oxford University Blavatnik School of Government. Since certain elements had to be modified to fit varying U.S. state conditions, we created another set of state-specific Stringency Indices that we call the Bentley State Stringency Index (BSI).

As the purpose of mitigation measures is to slow down the spread of infection, we introduced an exponential Mitigation Function on the transmission term in a time-varying SEIRD model. This Mitigation Function, incorporating the Bentley State Stringency Index (BSI), plays a crucial role in slowing down the progression of the disease, provided there is compliance. The latter is represented by an estimable Compliance Indicator (CI) that captures the average degree of compliance in each state. The Compliance Indicator thus modifies the effect of the BSI. It can allow the BSI to work at its full potential in reducing infection or it can progressively choke off completely the effect of mitigation policies as compliance by the public moves to zero. Sheikh et al. [18] outlined some indirect ways in which one may assess the degree of compliance such as by cell phone GPS data or traffic congestion and public transport usage. Our approach provides a data-based estimation of the degree of compliance for each state. To the best of our knowledge, our approach of employing a Mitigation Function with a tailored, state-specific Stringency Index and a Compliance Indicator has not been taken before.

The estimated Compliance Indicator was then simulated using varying values of the Stringency Index to bring the basic reproductive number R_0_ to less than 1 in each of the states studied. See [19, 20] for a history of the basic reproductive number and its complexities. A fundamental result in epidemiology is the “threshold” value of the basic reproduction number R_0_: “*There is a difference in epidemic behavior when the average number of secondary infections caused by an average infective during his/her period of infectiousness*, *called the* basic reproduction number, *is less than one and when this quantity exceeds one”* [21]. By focusing on the Mitigation Function with its two crucial components, the BSI and the Compliance Indicator, our analysis helps explain why some states have not been successful in controlling infections. In this study we demonstrate the efficacy of this metric from a public health and policy perspective by indicating the minimum level of compliance needed to control the epidemic, given a particular level of stringency. The visual tool created by us is simple enough to be understood by the public and can be used by policy makers to guide decisions as well.

## Data, variables and descriptive statistics

Our work is based on multiple data sources: the New York Times repository [22] of coronavirus data on GitHub, and state level data from the various state web portals [23–29]. Though these data sets begin from Jan 1 2020, COVID-19 infections were not apparent during the early period. Beginning from March 2 when the earliest observation is available, our data runs through July 31, 2020. Additional data on average household size and state population were gathered from the U.S. Department of the Census projections [30]. Within-household transmission is an important element for success in controlling the infection [31]. A study [32] of nearly 400 pregnant women in New York City did not find an association between infection and population density but did find a higher risk of COVID-19 infection due to increased household crowding.

We calculated daily cumulative infection cases (*confirmed infections)* from the daily numbers of new confirmed cases. Similarly, daily cumulative recovery (*recovery)* and daily cumulative death (*death)* were computed from daily numbers of recoveries and confirmed deaths. All three variables are from the New York Times repository [22] for each state. States may differ on how recovery is defined. For example, Texas calculates recoveries from those who are hospitalized by estimating the proportion of those who are hospitalized for no more than 32 days. To this number they add those who have not been hospitalized but have been infected with COVID-19 for 14 days [33]. Colorado bases recovery data on those discharged from COVID-19-related hospitalization [25]. For New Hampshire, recoveries are estimated from the resolution of COVID-19 fever without the use of fever-reducing medications and improvements in respiratory symptoms [24]. No definitions were released by New York, Arizona or New Mexico at the time the manuscript was completed. Due to the uneven data, there are a few missing daily entries that occur on different days in each state across the three different variables that we used: *daily confirmed infections*, *daily recovery* and *daily deaths*. These missing entries comprise 4% of our 2736 data points and were imputed by taking the average of the previous seven days. Since it takes the CDC coders that length of time to record COVID-19 deaths [34], the CDC uses a 7-day moving average to report new daily cases [35].

### Overview of the six states

According to WHO, to ensure that the testing rate is sufficient, the positivity rate should be between 3% to 5% [5]. As of August 13^th^, only New York, New Hampshire, New Mexico and Colorado were within this threshold. In mid-August, Arizona had the highest positivity rate of all 50 states (surpassed only by Puerto Rico with the highest possible, 100%). The trend of new cases per 100,000 shows a wide disparity in controlling the infection among these states. As seen from Table 1, New York which began with the highest rate of 1154.6 per 100,000 people, sank to the lowest (111.5) at the end of the period. On the other hand, Arizona started with 87.4 and rose to 1302.4. New Mexico and New Hampshire started with very similar rates (144.5 and 167.4 respectively), but by June New Mexico’s rate was almost twice that of New Hampshire.

**Table 1:** Overview of states.

State	Pos. R	New Cases per 100,000*				State imposed Any Restrictions				Average Household Size	Population
		April	May	June	July	April	May	June	July		
TX	14.99	85.6	124.8	330	900	Yes	Yes	Yes	Yes	2.86	28,995,881
NM	3.89	144.5	210.3	222.3	418.3	Yes	Yes	Yes	Yes	2.64	2,096,829
AZ	24.68	87.4	168.8	814.4	1302.4	Yes	Yes	Yes	Yes	2.69	7,278,717
CO	7.11	217.5	196.9	111.4	237.8	Yes	Yes	Yes	Yes	2.56	5,758,736
NH	2.03	167.4	233.1	113.3	76.9	Yes	Yes	Yes	Yes	2.46	1,359,711
NY	1.08	1154.6	325.9	115.0	111.5	Yes	Yes	Yes	Yes	2.60	19,453,561

Source: New York Times Repository of COVID-19 Data.

*This is obtained by dividing total new cases over the whole month by the state population and then multiplying by 100,000.

Abbreviations used: TX is Texas; NM is New Mexico; AZ is Arizona; CO is Colorado; NH is New Hampshire; NY is New York.

**Fig 1:**
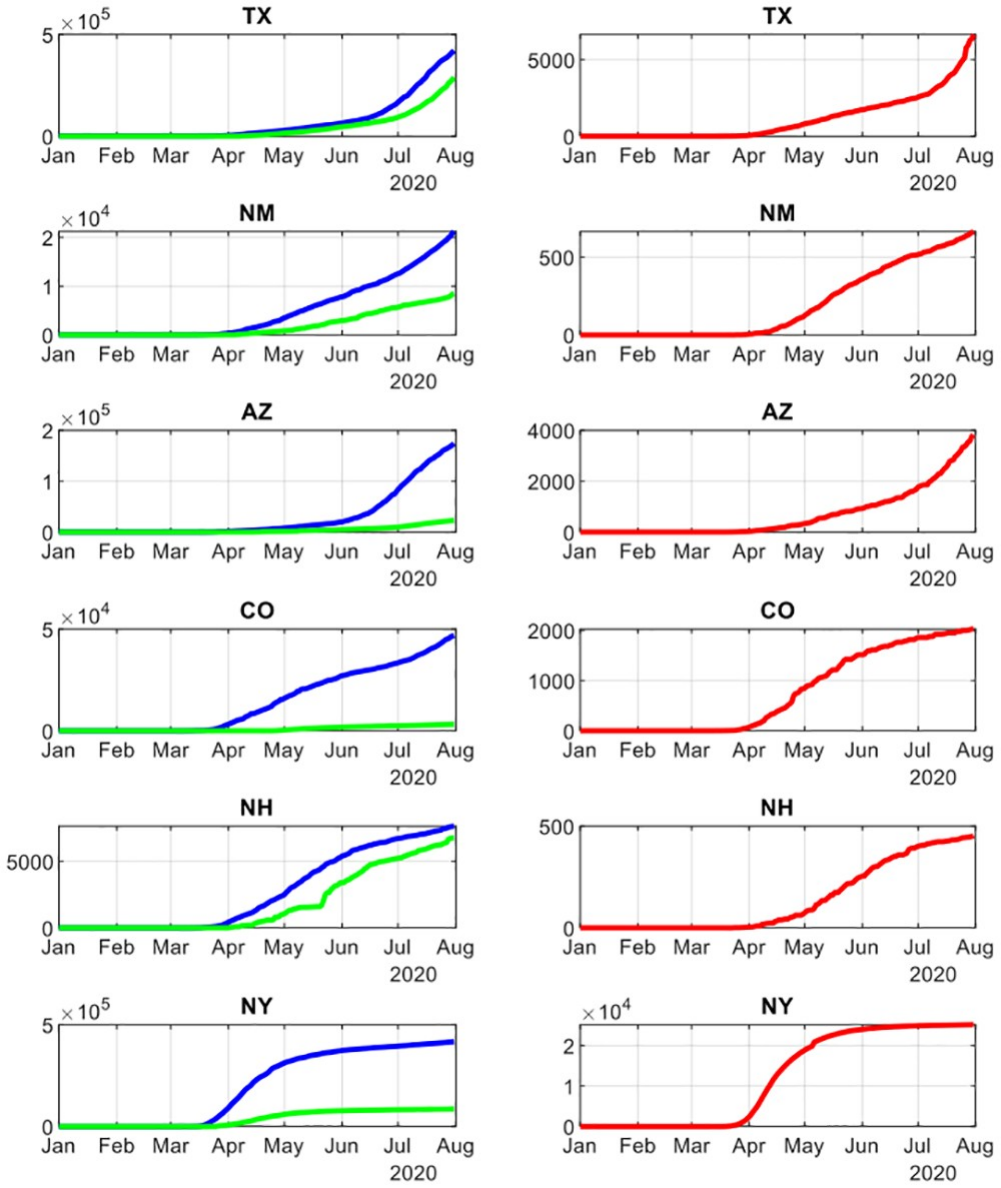
Time series plots for cumulative infection recovery and death for 6 states. **Legend**: Time series plot for all six states on cumulative confirmed infections, recovery and death. The left column has the cumulative infection (blue) and recovery cases (green) for each state from January to July 2020. The right column has the cumulative deaths for each state for the same period. The varying patterns between the states show up clearly.

These varying trends of infections and recoveries in each state are shown in Fig 1. In some states like Texas, Arizona and New Mexico, the infection rate has accelerated from a previously slower rate of increase. However, in each state there is a clear point of inflection that occurred in June. On the other hand, the remaining three states, most noticeably New York, began entering a phase where the infection was flattening at different rates. Investigating this disparity is the objective of this paper.

### The stringency index

A Stringency Index was calculated for each state by modifying the Oxford Index as shown in Appendix B in S1 Appendix. Three items that could not be included by us were restrictions that either were not applicable at the State level or were not applicable to the U.S. in general. These were restrictions on internal travel controls, international transportation, and public officials commenting or coordinating campaigns. However, we needed to add four important restrictions applicable to the U.S. that were not included in the Oxford Index. These were the wearing of face masks, social or physical distancing of 6 feet, nursing home visiting restrictions as per CDC guidelines [36–38], and state border restrictions. Stutt, Retkute, Bradley, Gilligan, & Colvin [39] showed the effectiveness of wearing face masks in managing the COVID-19 pandemic. It may be noted that instead of the term “social distancing”, some are advocating the term “physical distancing” to clarify to clarify that social connectivity is to be encouraged while yet maintaining physical distancing [40].

Following the methodology adopted by the Oxford University Blavatnik School of Government (see Appendix A in S1 Appendix) the Bentley Stringency Index (BSI) was calculated for each state for the entire period. What do these BSI numbers mean? The two extremes of the BSI are 0 where there are no restrictions, and 10 where all the restrictions given in Table 2 (Appendix A in S1 Appendix) apply to the entire state at the maximum of the scales applicable to that category. The actual BSI will be a combination of the different elements and the scale at which they are applied. For instance, if face masks are recommended but not required, the level of the variable H6 (Appendix A in S1 Appendix) becomes 1 instead of the maximum of 2 and the BSI will go down. Therefore, a particular BSI number cannot point to a unique combination of mitigation measures but may be consistent with different combinations of restrictions whether applied to the entire state or to targeted areas.

To further clarify the significance of the BSI, we illustrate it from our calculations using the state data from New York and Arizona, two states with very different success in controlling their infection rates. On the 25 April, the BSI for New York was 6.47 but the next day, 26^th^ April, the BSI jumped to 7.03 because the testing policy was refined to say that anyone showing COVID-19 symptoms should be tested. In Arizona, the BSI on March 30 was 3.75 and the next day it rose to 4.72. The big jump was due to the introduction of new stay at home requirements and limits on public transportation. Again, in Arizona, on May 1, there was a one day drop in the BSI from 5.0 to 4.4 reflecting the removal of the stay at home order.

**Fig 2:**
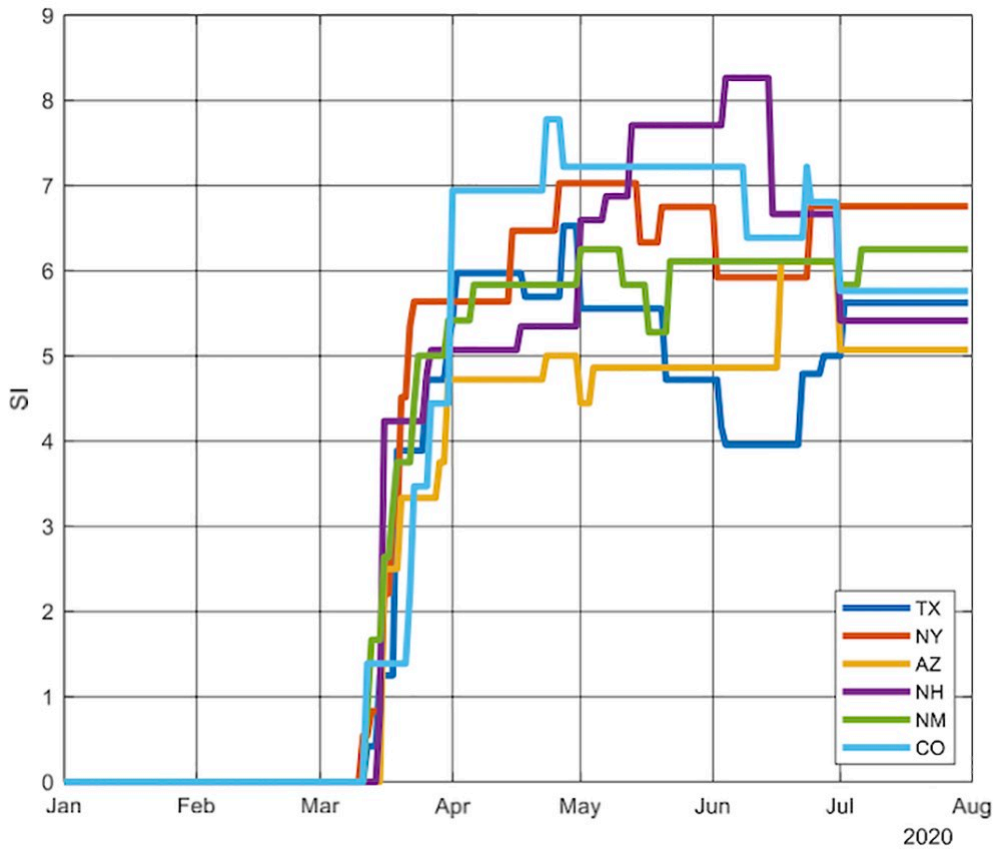
Path of mitigation measures (Bentley Stringency Index) over time in each state. **Legend**: A comparison of the degree of stringency of mitigation measures by each state shows that though the states all responded by introducing mitigation measures, there are substantial variations between states.

The movement of the BSI for each state is shown in Fig 2. Notice that the six states began mitigation interventions around mid-March but subsequently they diverged in terms of timing and extent. By April, Colorado had the highest BSI, whereas Arizona had the lowest. New York slackened restrictions in June and was slightly below New Mexico, but from mid-April to May, New York was more restrictive than New Mexico.

### The model

**Fig 3:**
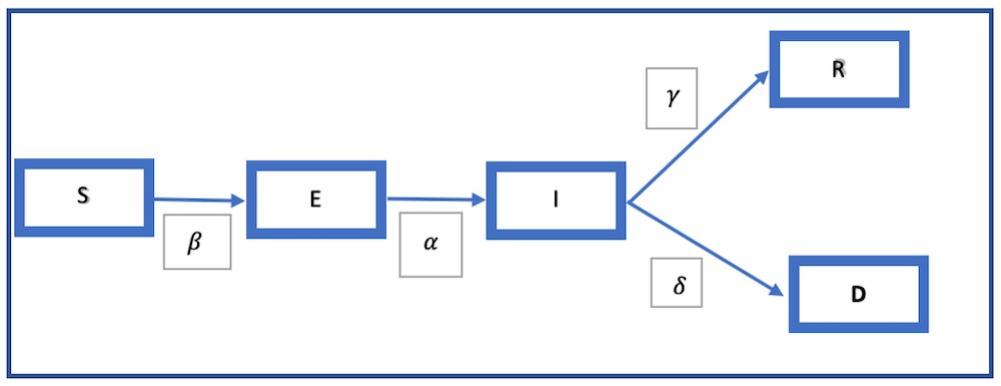
The SEIRD model flow chart. **Legend**: This model shows how, beginning from S, the susceptible population moves from being exposed E, to infected I, and then to either recovery R, or death D, the movement being regulated by different parameters.

The model that we adopt is based on the SEIRD (Susceptible, Exposed, Infected, Recovered or Died) model that has been developed by Weitz and Dushoff [41] Loli and Zama [42] and Lattanzio and Palumbo [43]. Diagrammatically it can be shown as follows Fig 3.

The structure of this model that is used in the literature can be described by the following equations:

$$\frac{dS}{dt}=-\beta \frac{S∙I}{N}$$ (1)

$$\frac{dE}{dt}=\beta \frac{S∙I}{N}-\alpha E$$ (2)

$$\frac{dI}{dt}=\alpha E-\gamma I-\delta I$$ (3)

$$\frac{dR}{dt}=\gamma I$$ (4)

$$\frac{dD}{dt}=\delta I$$ (5)

Following Lolli and Zama [42], we have the compartments S, E, I, R and D where S is the susceptible group, E consists of those who are infected but may not be infectious, I is the infectious group, while R and D respectively consist of those who recovered or died.

S(t) comprises the total state population at time t after subtracting for those who were exposed or infected, and those who recovered or died. E(t) is approximated by the cumulative number of people tested at time t, and I(t) is the cumulative confirmed positive cases at time t. The variables S(t), E(t) and I(t) were taken from the Covid Tracking Project [23] and are observed. SI(t), the Bentley Stringency Index that has been calculated as explained earlier is the average measure of the degree of stringency of a state’s mitigation policies.

The term *β* (though with slightly different names in the literature) is often called the transmission rate of infection or the rate at which two specific individuals come into effective contact per unit of time [44, 45]. Specifically, *β* is the product of the Contact Rate (average number of contacts per person per unit of time) * Transmission Probability or probability of disease transmission in a contact. *α* is the incubation rate, and 1/*α* is the average period (days) of moving from E to I. The average infectious period (days) for the infectious group I is 1/*γ*. For instance, if *γ* = 0.2, then 1/*γ* = 5 days means that with an infectious period of 5 days, 20% of patients recover each day. *γ* denotes the recovery rate for those infected and who have recovered. They move from I to R. *δ* is the death rate corresponding to a movement from I to D. *α*_*t*_, *β*_*t*_, *γ*_*t*_, *δ*_*t*_, *θ*_*t*_ are estimated parameters emerging from the model structure. This model assumes that there is no reinfection and so eliminates movement from R to S. In addition, due to the short period under consideration for epidemics, the population is assumed to be constant with equal birth and death rates.

We extend this SEIRD model by explicitly modeling two important drivers of *β*, the transmission rate. In the epidemiological literature, it is acknowledged that *β* can depend on factors like age, living conditions and behavioral interventions such as the closing of theaters, schools and staggering of office hours as happened during the 1918 pandemic [11, 12, 45]. Thus the standard *β* in the literature is a kind of “black box” containing a complex of factors affecting *β* and the transmission of disease. We investigate two important factors that influence *β*: mitigation policies that the BSI captures, and the compliance to these by the public. The latter is being increasingly discussed in the media as being crucial to the success of the various mitigation measures. On Aug 5 2020, in a virtual symposium hosted by the Harvard University’s T.H. Chan School of Public Health, Dr. Fauci, Director of the National Institute of Allergy and Infectious Diseases, explained that it was due to the difference in the states’ mitigation measures and the different ways in which the public has complied with these measures that the U.S. is having difficulty in controlling the pandemic. In the words of the Harvard Gazette, “In addition, he (Dr. Fauci) said, state reopening plans proceeded at different paces. Some states reopened slowly, similar to the pace of European nations, while others went much faster. Another variable, he said, was the extent to which residents of different states adhered to reopening guidelines, with some following recommendations while others ignored the restrictions, sometimes in notably large groups” [46].

To operationalize these two factors that drive *β* we explicitly formulate a Mitigation Function that acts to reduce the transmission of disease. This *Mitigation Function* has two major components: the *Bentley Stringency Index* (BSI) and what we call the *Compliance Indicator* (CI). The third component, average household size, is taken as constant over the period under consideration. The BSI has been calculated daily for each state while the Compliance Indicator is estimated from our adaptive computing procedure.

We therefore propose the following formula for *β*:

$$\beta =\beta_{0}∙{\frac{1}{e}}^{k∙SI∙\theta }$$

*β*_0_ connotes the transmission without policy intervention, *e*^*k*·*SI*·*θ*^ is defined as the Mitigation Function, k is 1/(average household size) and is a fixed parameter for each state taken from the US Census [30]. *SI* is the stringency index, and *θ* is the Compliance Indicator. *β* will decrease at an exponential rate of ${\frac{1}{e}}^{k∙SI∙\theta }$.

To incorporate the transmission factor in a time-varying model, we extended *β* to a time-dependent format *β*_*t*_ in our model for estimation purposes.


$$\beta_{t}=\beta_{0}∙{\frac{1}{e}}^{k∙{SI}_{t-h}∙\theta_{t}}$$

The time-varying Mitigation Function is: $e^{k∙{SI}_{t-h}∙\theta_{t}}$ where, *SI*_*t*_ is the stringency index at time t. The time lag introduced by a delay in policy implementation is denoted by *h*. We assume a modest policy lag of 1 day in our model using daily data. *θ*_*t*_ is the Compliance Indicator at time t. The thrust of the model is the estimation of the Compliance Indicator *θ*_*t*_ and in the process, it also estimates the other unknown parameters: *β*_0_, *α*, *γ*, and *θ* that have been defined above.

Note that the Compliance Indicator (CI) can vary from 0 (no one complies) to a theoretical maximum of 1 (everyone complies). When the CI = 0, the BSI index has no effect irrespective of its value and the model collapses to the standard model where the mitigation efforts do not affect *βt*. On the other hand, the BSI can also vary from 0 (no restrictions) to a theoretical maximum of 10 (akin to a total lockdown). This 0 to 10 scale follows the 0 to 100 scale used in the GRSI by Oxford University. When BSI = 0, the model again collapses to the standard model. When BSI is greater than 0, then the effect on *βt* will depend also on the Compliance Indicator. Even if BSI is at its maximum, a low CI will reduce the Mitigation Function. In other words, it is both BSI and the Compliance Indicator that will determine (given the household size that varies by state) the power of the Mitigation Function which affects the transmission of the disease. Incorporating this extension, our model structure is as follows.


$$\frac{dS}{dt}=-\beta_{0}∙e^{-k∙{SI}_{t-h}∙\theta_{t}}∙\frac{S_{t}∙I_{t}}{N}$$

$$\frac{dE}{dt}=\beta_{0}∙e^{-k∙{SI}_{t-h}∙\theta_{t}}∙\frac{S_{t}{∙I}_{t}}{N}-\alpha E_{t}$$

$$\frac{dI}{dt}=\alpha E_{t}-\gamma I_{t}-\delta I_{t}$$

$$\frac{dR}{dt}=\gamma I_{t}$$

$$\frac{dD}{dt}=\delta I_{t}$$

## Methodology and results

To estimate the parameters of our proposed model, we use numerical analysis methods and statistical approaches with COVID-19 data from six states beginning from March 2020 to July 2020. In this computing process, we first develop the difference equation system as per the following system:

$$S\left(t+1\right)=S\left(t\right)-\beta_{0}∙e^{-k∙{SI}_{t-1}∙\theta_{t}}∙\frac{S_{t}∙I_{t}}{N}$$

$$E\left(t+1\right)=E\left(t\right)+\beta_{0}∙e^{-k∙{SI}_{t-1}∙\theta_{t}}∙\frac{S_{t}{∙I}_{t}}{N}-\alpha E_{t}$$

$$I\left(t+1\right)=I\left(t\right)+\alpha E_{t}-\gamma I_{t}-\delta I_{t}$$

$$R\left(t+1\right)=R\left(t\right)+\gamma I_{t}$$

$$D\left(t+1\right)=D\left(t\right)+\delta I_{t}$$

Then, we develop the overall error function: *Error*(*t*) = max{*Er*_*S*_(*t*), *Er*_*I*_(*t*), *Er*_*R*_(*t*), *Er*_*D*_(*t*)}, where ${Er}_{S}\left(t\right)=\left|\hat{S}\left(t\right)-S\left(t\right)\right|;{Er}_{I}\left(t\right)=\left|\hat{I}\left(t\right)-I\left(t\right)\right|;{Er}_{R}\left(t\right)=\left|\hat{R}\left(t\right)-R\left(t\right)\right|$; and finally, ${Er}_{D}\left(t\right)=\left|\hat{D}\left(t\right)-D\left(t\right)\right|$.

As is commonly used, we take the absolute difference between $\hat{S}\left(t\right)$ and *S*(*t*) to denote the error *Er*_*S*_(*t*) at time t. We use a similar process to define the errors for Infection (*Er*_*I*_(*t*)), recovery (*Er*_*R*_(*t*)) and death (*Er*_*D*_(*t*)) at time t.

$\hat{S}\left(t\right),\hat{I}\left(t\right),\hat{R}\left(t\right),\hat{D}\left(t\right)$ are the model estimations at time t for susceptible cases, confirmed infections, recovered individuals and those who died. Correspondingly, *S*(*t*), *I*(*t*), *R*(*t*), *D*(*t*) are the observed data for each compartment at time t. The estimated value**s** of the parameters at time t giving the minimum of *Error*(*t*), were found by applying the interior-point algorithms with dynamically modified constraints on the parameter estimations. Parameters ($\hat{S},\hat{I},\hat{R},\hat{D}$ in all six states) were estimated, and predicted values found by the 4^th^ order Runge-Kutta method using MATLAB 2020a.

The model provides insights into the relationship between three crucial factors in controlling an epidemic: the Bentley Stringency Index (embodying the policy measures recommended or required by Federal or State authorities), the Compliance Indicator (embodying the extent of compliance by the public to these policy measures) and *R*_0_ the basic reproductive rate (defined in the literature as the average number of people an infectious person will infect assuming that the rest of the population is susceptible). It is important to note that though the Mitigation Function has the two variables, BSI and the CI entering as a product, the Compliance Index is the only one endogenously determined and estimated from the model. The BSI is pre-determined and has been built exogenously. Thus, it is possible at any point of time to identify the Compliance Index independently of the BSI.

Having the estimated parameters, the next step explores the interaction of the time-varying values of the Stringency Index and the Compliance Index in each state with the movement of the infection rates.

To examine this, we did two sets of simulations to explore the interacting effect of BSI and CI on *R*_0_. Both simulations are based on the formula above, with fixed parameters of an average household size of 2.6 individuals [31], *γ* = 0.2 [47, 48] and *δ* = 0.032 [1]. *β*_0_ = 2 was taken from the range of transmission rates (1.5 to 3.5) estimated by Statista [49].

### Simulation 1: Comparing the Oxford and Bentley stringency indices

For both simulations, we used our proposed formula for *β* and calculated the movement of *R*_0_ using both the Bentley and the Oxford indices. The basic reproduction number *R*_0_ is:

$$R_{0}=\frac{\beta }{\gamma +\delta }=\beta_{0}∙\frac{1}{e^{k∙SI∙\theta }}∙\frac{1}{\gamma +\delta }$$

For an epidemic to die out, *R*_0_ must be less than one.

**Fig 4:**
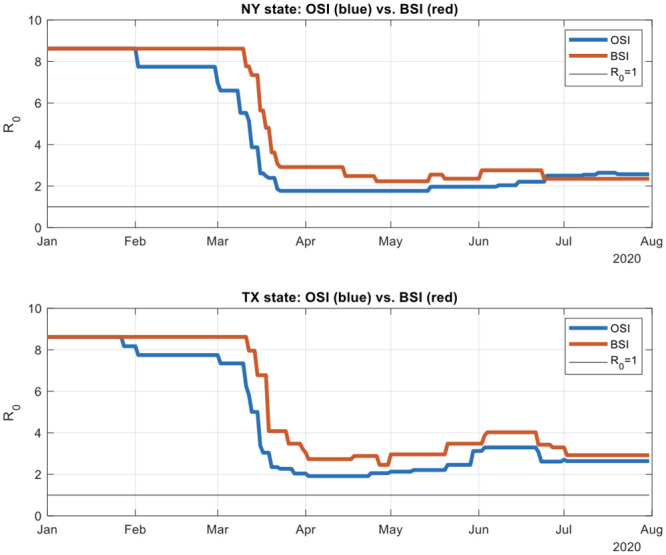
Comparison of Oxford Stringency Index (OSI) and Bentley Stringency Index (BSI). **Legend**: This graphs shows the simulated R_**0**_ by using New York and Texas as examples. The simulation period was from January to August 2020. The BSI gives a slightly more conservative result than the OSI.

We ran the comparative simulations with fixed parameters, including the Compliance Indicator on all 6 states. The results (Fig 4) are reported for New York and Texas. Results for other states, not reported in the interest of space, are available on request. Comparing the two Stringency Indices, the Bentley Stringency Index is overwhelmingly (with a few exceptions) more conservative in that the simulated *R*_0_ is higher than that obtained by using the Oxford Index.

These simulations gave us further confidence about using the Bentley Stringency Index which has been constructed using policy conditions specific to each state. We were able to start with the first statewide announcement regarding mitigation measures [25, 27].

### Simulation 2: Interaction of compliance indicator and *R*_*0*_ at different levels of BSI

**Fig 5:**
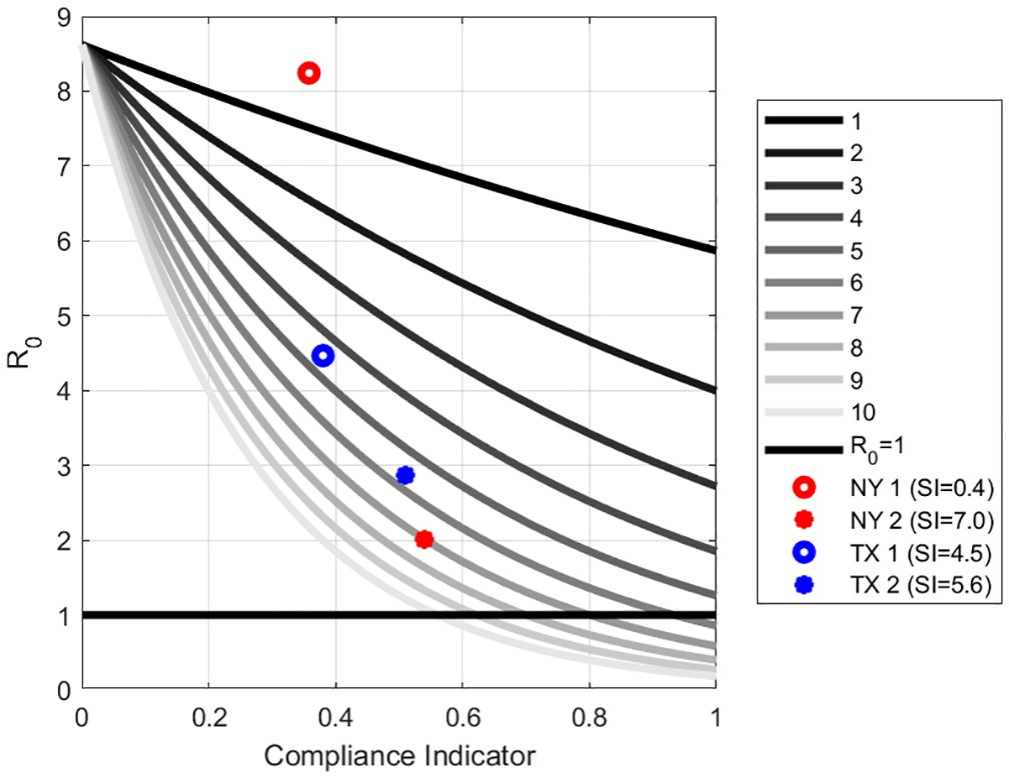
The combination of Stringency and Compliance needed at various levels of R_0_. **Legend**: This simulation tool shows the various combinations of policy measures and degrees of public compliance that will be sufficient to bring R_0_<1 thus bringing the pandemic under control. R_0_ is indicated by the bold black line which divides the graph into the “undesirable” portion (above) and the “desirable” portion (below). We have simulated where New York and Texas were for two different periods as they moved closer to the desirable portion.

Next, we compared New York and Texas by simulating the Compliance Indicator and *R*_0_ at different levels of the BSI, using the same formula as used in the first simulation. See Fig 5.

In Fig 5, the vertical axis is *R*_0_ while the horizontal axis shows the range of the Compliance Indicator. Each grey scale line shows the relationship between *R*_0_ and the Compliance Indicator at different levels of the Stringency Index. The maximum Stringency Index is 10 and is shown by the lowest line. The horizontal line denotes *R*_0_ = 1 which is accepted in the literature as the threshold above which the epidemic will keep spreading. The lower part of the graph can be denoted as “desirable” and the upper portion as “undesirable”. When *R*_0_ is less than one the epidemic will die out.

Our simulations show the different levels of compliance that would be compatible with different degrees of stringency in order for *R*_0_ to go below the threshold. For instance, when the BSI is at 1, then even if the Compliance Indicator is at the maximum, *R*_0_ will not be lower than one. On the other hand, with the highest value of the BSI, the Compliance Indicator has to be at least 25% for *R*_0_ to be at the threshold.

Since Fig 5 is based on the average household size in the U.S., it must be remembered that when the household number increases, then to reach the same *R*_0_ level, the combination of the Stringency Index and the Compliance Indicator has to be at higher levels.

For instance, New York began high in the “undesirable” portion as the higher red dot indicates. That was during Day 65 (March 5th) to Day 75 (March 15th). However, between Day 120 (April 29^th^) to Day 130 (May 9^th^), New York moved to closer to the “desirable” portion of the graph as the lower red dot shows. Similarly, Texas (blue dot) also moved from an undesirable point between Day 170 (June 18^th^) to Day 180 (June 28^th^) to being closer to the “desirable” portion during the period Day 190 (July 8^th^) to Day 200 (July 18). Neither state was, however, successful in crossing into the desirable portion of the graph.

**Fig 6:**
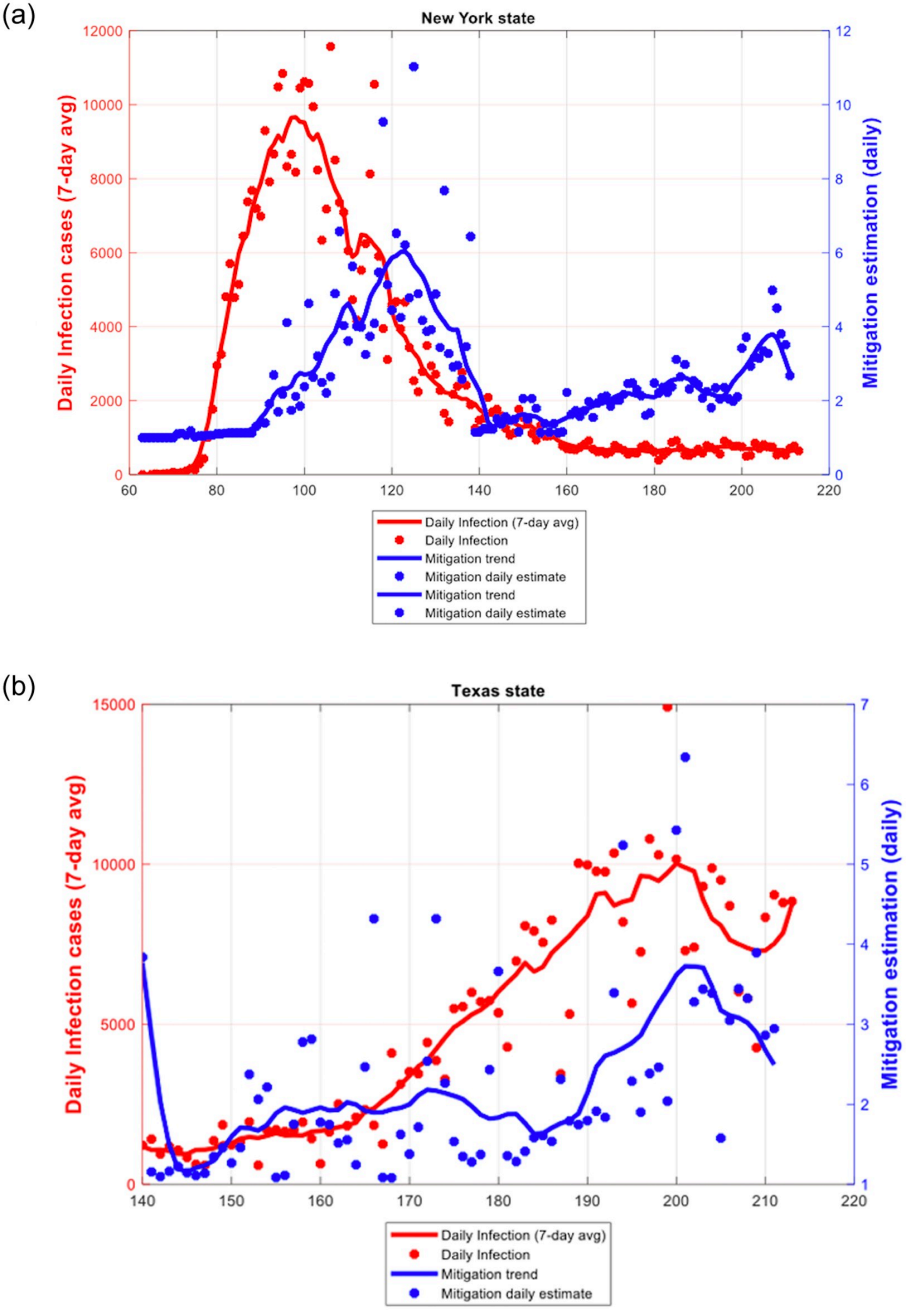
a. New York: Plots of mitigation function estimation (daily) vs daily infections (7-day average). Legend: This figure for New York shows the pattern of the relationship between the observed daily infection cases (red dots) with the mitigation function (blue dots). The red line is the 7-day average of confirmed daily infection cases. The blue line is the trend line of the mitigation function obtained by spline smoothing. b. Texas: Plots of mitigation function estimation (daily) vs daily infections (7-day average). Legend: This figure for Texas shows the pattern of the relationship between the observed daily infection cases (red dots) with the mitigation function (blue dots) as measured by the BSI. The red line is the 7-day average of confirmed daily infection cases. The blue line is the trend line of the mitigation function obtained by spline smoothing.

Fig 5 can be of use to guide policy regarding the extent of mitigation measures and compliance needed by the public. Let us illustrate what we mean by going back to the two states we showed in Fig 5, namely New York and Texas. In Fig 6a and 6b we show plots of the estimated mitigation function against daily infections.

In Fig 6a for New York, the inverse relation between the Mitigation Function (blue line) and daily infections (red line) can be clearly seen. It must be remembered that the Mitigation Function captures only a portion of all the factors that drive *β*, the transmission rate. When the infection was raging, the Mitigation Function was increasing and we find that the BSI increased from, essentially, 0 to 0.8 with a mean value of 0.358 during the 10-day period March 5^th^ to March 15^th^ (Days 65 to 75). This was because New York restricted public gatherings, cancelled public events and recommended against nursing home visits. The mean estimated CI was 36%. This placed New York in the upper “undesirable” portion of the graph in Fig 5. When the state initiated the face mask requirement around April 15^th^ (Day 106) and the testing policy was introduced and defined on April 26 (Day 117), infections, which had hit a peak, began a trajectory downwards until it plateaued around mid-June. By the beginning of May the infection had abated, and the mean values of BSI and CI were 7.028 and 54% respectively during the 10-day period between April 29^th^ and May 9^th^. This trend corresponded with the announcement of New York’s COVID-19 Testing Policy. With this combination of BSI and Compliance Index, New York moved closer to the lower “desirable” range (Fig 5) as the infection rate came more under control.

For Texas, on May 20^th^ (day 141 in the figure) Governor Abbott signed an executive order on Phase III reopening, to allow more businesses to reopen and to reduce restrictions on gatherings. The Mitigation function decreased accordingly. Note in Fig 6b that as the Mitigation Function decreased with the relaxation of state restrictions, COVID-19 infections also began to climb.

Further, between Day 170 (June 18^th^) to Day 180 (June 28^th^), the BSI increased from 3.9 to 5.0 with a mean value of 4.5. The mean value of the estimated CI is 31%. Even though the state had increased the mitigation policies, it was not enough. The fact that the compliance also is far too low can be seen with a little thought experiment using Fig 5. Suppose the BSI were 5 (more stringent), then the compliance indicator would need to be at least 50% for the infection to die out as Texas moves to the desirable lower portion of the graph. On the other hand, with this low level of compliance, it can be confidently predicted that the infection will rise. In fact, in the following weeks between June 22 and July 17 the confirmed COVID-19 cases reached 14,916, the highest one-day mark for Texas.

Our results also indicate that when stringency measures are constant, changes in the Compliance Indicator is associated with changes in the infection rates. In Texas, from July 2nd to July 18th (Day 184 to 200 since Jan 1^st^), the BSI stayed constant at 6.18. On the other hand, the estimated CI showed a dramatic 10-day average increase from approximately 24% to 44%.On June 4, Texas reduced restrictions on gatherings by allowing for indoor assemblies such as at places of worship, among local government operations, child-care services and recreational sports for youths and adults. The daily infections during the corresponding period reached a peak and then began to slowly decrease from the end of July 2020. This is clearly visible in Fig 6b.

To examine the sensitivity of the estimated compliance indicator from our model, we conducted three sets of sensitivity analyses using New York as a test case. With a 0%-5% random perturbation (increase or decrease) of daily infection data, we did N = 1000 simulations on the Compliance Indicator, household size and the Bentley Stringency Index. The results are in the S1 Table and show acceptable 90% confidence levels.

## Discussion and conclusion

The objective of this paper was to examine how mitigation policies and compliance to these polices can combine to advance or frustrate the fight against the COVID-19 pandemic. Our concern arose from the observation that states with similar community mitigation measures were experiencing very divergent trends in infection rates. To the best of our knowledge, there is no epidemiological model that can help us understand this phenomenon. In this paper we propose a simple modification to bring both mitigation policies and compliance into a standard epidemiological model and, in exploring their interaction, see the minimum levels of each that would lead each state studied to a point where the epidemic would die out.

To accomplish our purpose, we use a standard SEIRD model and then explicitly incorporate two factors that are already present in the “black box” that is *βt*: mitigation measures and public compliance. In doing this, we build upon the work of Kurcharski et al. [8] where they suggest that a combination of methods (testing, tracing, physical distancing, self-isolation and quarantine) may be needed to reduce effective transmission so that the epidemic is contained. We go further by taking a vector of mitigation policy measures and encapsulating them into a Bentley Stringency Index (BSI) similar to that built by Blavatnik School of Government of the University of Oxford. Building on and extending their methodology, we create state stringency indices by incorporating directives like wearing face masks, restrictions on nursing home visits that are appropriate to U.S. states to different degrees.

The best of mitigation measures need to be actually followed if they are to be successful. To capture public compliance, a state-specific Compliance Indicator was estimated from the model using the daily COVID-19 data from each state. In this context, Cano et al. [50] have modelled scenarios with different levels of what they have termed social distancing. However, this term is also used by them interchangeably with lockdown. They show that the less seriously the public takes the lockdown measures, the longer the epidemic will take to resolve and the number of deaths will increase. By quantifying public compliance through the Compliance Indicator, we build the Mitigation Function that encompasses both the BSI and the CI. We demonstrate the association of the movement of infections with movements in the Mitigation Function, ceteris-paribus. Thus, it is the combined effect of the mitigation measures and the compliance that is key. Without either, there can be no containing the pandemic.

Compliance by the public to mitigation measures is largely exogenous in democratic societies. However, by coordinated and effective public information campaigns, through example, by helping people understand that obeying these restrictions is crucial to reclaiming their lives, compliance may be enhanced. In this regard, Arriola and Grossman’s work, though in the context of Africa, may be interesting [51]. They wanted to see how the social identity of individuals could affect their compliance with advice from public health officials. In the U.S., some states have adopted the “stick” approach; California cut off power to those who were defying restrictions [52]. However, if the public understands that it is in their own self-interest, the degree of compliance can be increased with their cooperation.

We have also contributed by suggesting a practical, real-time visual policy tool that can be used flexibly not only to monitor the progress in controlling the disease but also to adjust policy in a dynamic fashion. It can be used to support decisions in adjusting mitigation policies by taking into consideration the level of public compliance as well. This tool is also simple enough to be used to educate the public on the importance of compliance.

The chief limitation of our analysis is a problem that all researchers on COVID-19 have to contend with at this time with an ongoing deadly and fast-moving pandemic. Even though we had only 4% of our observation points that were missing or questionable, there are concerns regarding the overall quality of the data [53]. In addition, a critical issue that has emerged is the under-reporting of cases with continued difficulties in getting tested. We have conducted a sensitivity analysis by adding a random percentage increase of up to 5% to the daily observed infection cases and then examining the variation in the estimated result with regard to both compliance and mitigation (see S1 and S2 Figs). As expected, this does affect the precision of the estimates to varying degrees in different states. Though this is a shortcoming that we are unable to overcome, we do not believe that this affects the main thrust of our paper which is the importance of considering the dual impact of mitigation policies and compliance by the public on controlling the pandemic. The transmission factor β, has hitherto been a “black box”. What we have done is taken the first step to model two important drivers of β.

Our work has opened up several avenues of future research and we give here only a sample of possibilities. With a relatively scant literature on compliance, we hope our work will stimulate more research on this. An important contribution that we intend to take up in a future study would be an exploration of the optimal path of the Compliance Indicator over time. Our method of using the Mitigation Function in SEIRD can be applied to other epidemiological models as well. In addition, this methodology can also be translated to other countries, thereby providing another tool to the authorities in combating this pandemic. We have also made the first step in attempting to quantify the factors that go into the “black box” of *β* and hope that our work will stimulate further exploration.

## Supporting information

S1 Table 1000 simulations of daily infections, household size and Bentley Stringency Index on the Compliance Indicator in the case of New York. Legend: This table shows the effect on the Compliance Indicator by perturbing 3 sets of numbers at a time: the daily infection, household size, k and the Bentley Stringency Index. • N refers to the number of simulations done. • std_t is the standard deviation of 1000 simulations at time t. • Mean of std_t is the average of all the 1000 standard deviations obtained. • The 90% Confidence Interval is obtained by sorting all the std_t in ascending order and then computing the 5^th^ and 95^th^ percentiles.
(TIF)

S1 Fig Sensitivity analysis: NY 90% confidence band for estimated compliance rate and trend. Legend: This figure shows the simulation on NY observed infection cases from Mar to July. A random inflation rate (uniformly from 0% to 5%) was applied to the observed infection cases for each day. The daily compliance rate was estimated from the algorithm. With N = 1000 simulation, the 90% confidence band for the daily compliance rate was shown in the figure, as well as the 7-day average (blue dots) and spline smoothing trend (blue line).
(TIF)

S2 Fig Sensitivity analysis: NY 90% confidence band for estimated mitigation and trend. Legend: This figure shows the simulation on NY observed infection cases from Mar to July. A random inflation rate (uniformly from 0% to 5%) was applied to the observed infection cases for each day. The daily compliance rate was estimated from the algorithm. With N = 1000 simulation, the 90% confidence band for the daily mitigation function from the daily compliance rate was shown in the figure, as well as the 7-day average (blue dots) and spline smoothing trend (blue line).
(TIF)

S1 Appendix (DOCX)

## References

[1] COVID-19 in the USA at Johns Hopkins University. https://coronavirus.jhu.edu/. [Accessed August 25, 2020].

[2] Hasell J, Ortiz-Ospina E, Mathieu E, Ritchie H, Beltekian D, Macdonald B, et al. Coronavirus (COVID-19) testing. Our World in data. https://ourworldindata.org/coronavirus-testing#the-positive-rate-are-countries-testing-enough-to-monitor-their-outbreak. [Accessed August 25, 2020]. 10.1038/s41597-020-00688-8 PMC7545176 33033256

[3] World Health Organization. WHO Update on COVID-19 Full Press Briefing Video. https://www.youtube.com/watch?v=jMCzQhqOzh4. [Accessed March 30, 2020].

[4] United Nations, Department of Economic and Social Affairs, Population Division. World Population Prospects: The 2019 Revision. New York: United Nations. https://worldpopulationreview.com/countries/united-states-population. [Accessed August 25, 2020].

[5] Which U.S. States Meet WHO Recommended Testing Criteria? At Johns Hopkins University. https://coronavirus.jhu.edu/testing/testing-positivity. [Accessed May 12, 2020].

[6] Do TS, Lee YS. Modeling the spread of Ebola. Osong public health and research perspectives. 2016 Feb 1;7(1):43–8. View Article • Google Scholar doi: 10.1016/j.phrp.2015.12.012 26981342 PMC4776269

[7] Anderson RM, Heesterbeek H, Klinkenberg D, Hollingsworth TD. How will country-based mitigation measures influence the course of the COVID-19 epidemic?. The lancet. 2020 Mar 21;395(10228):931–4. View Article • Google Scholar doi: 10.1016/S0140-6736(20)30567-5 32164834 PMC7158572

[8] Kucharski AJ, Klepac P, Conlan AJ, Kissler SM, Tang ML, Fry H, et al. Effectiveness of isolation, testing, contact tracing, and physical distancing on reducing transmission of SARS-CoV-2 in different settings: a mathematical modelling study. The Lancet Infectious Diseases. 2020 Oct 1;20(10):1151–60. View Article • Google Scholar doi: 10.1016/S1473-3099(20)30457-6 32559451 PMC7511527

[9] Hellewell J, Abbott S, Gimma A, Bosse NI, Jarvis CI, Russell TW, et al. Feasibility of controlling COVID-19 outbreaks by isolation of cases and contacts. The Lancet Global Health. 2020 Apr 1;8(4):e488–96. View Article • Google Scholar doi: 10.1016/S2214-109X(20)30074-7 32119825 PMC7097845

[10] Prem K, Liu Y, Russell TW, Kucharski AJ, Eggo RM, Davies N, et al. The effect of control strategies to reduce social mixing on outcomes of the COVID-19 epidemic in Wuhan, China: a modelling study. The Lancet Public Health. 2020 May 1;5(5):e261–70. View Article • Google Scholar doi: 10.1016/S2468-2667(20)30073-6 32220655 PMC7158905

[11] Hatchett RJ, Mecher CE, Lipsitch M. Public health interventions and epidemic intensity during the 1918 influenza pandemic. Proceedings of the National Academy of Sciences. 2007 May 1;104(18):7582–7. View Article • Google Scholar doi: 10.1073/pnas.0610941104 17416679 PMC1849867

[12] Bootsma MC, Ferguson NM. The effect of public health measures on the 1918 influenza pandemic in US cities. Proceedings of the National Academy of Sciences. 2007 May 1;104(18):7588–93. View Article • Google Scholar 10.1073/pnas.0611071104 PMC1849868 17416677

[13] Teslya A, Pham TM, Godijk NG, Kretzschmar ME, Bootsma MC, Rozhnova G. Impact of self-imposed prevention measures and short-term government-imposed social distancing on mitigating and delaying a COVID-19 epidemic: A modelling study. PLoS medicine. 2020 Jul 21;17(7):e1003166. View Article • Google Scholar doi: 10.1371/journal.pmed.1003166 32692736 PMC7373263

[14] Hale T, Atav T, Hallas L, Kira B, Phillips T, Petherick A, et al. Variation in US states responses to COVID-19. Blavatnik School of Government. 2020 Aug 17. View Article • Google Scholar

[15] Hale T, Petherick A, Phillips T, Webster S. Variation in government responses to COVID-19. Blavatnik school of government working paper. 2020 May 27;31:2020–11. View Article • Google Scholar

[16] Jayatilleke AU, Dayarathne S, de Silva P, Siribaddana P, Abeygunawardana RA, Nieveras O, et al. COVID-19 case forecasting model for Sri Lanka based on Stringency Index. medRxiv. 2020 Jan 1. View Article • Google Scholar

[17] Barberia LG, Cantarelli L, Claro ML, Rosa IS, da Silva Pereira F, Zamudio M. Confronting the COVID-19 Pandemic: Brazilian Federal and Subnational-Government Responses, Technical Report on Social Distancing Stringency (SDS) 1.0. Tech. rep. 2020 Apr 8. View Article • Google Scholar

[18] Sheikh A, Sheikh Z, Sheikh A. Novel approaches to estimate compliance with lockdown measures in the COVID-19 pandemic. Journal of global health. 2020 Jun;10(1). View Article • Google Scholar doi: 10.7189/jogh.10.010348 32426117 PMC7211415

[19] Heesterbeek JA. A brief history of R 0 and a recipe for its calculation. Acta biotheoretica. 2002 Sep;50(3):189–204. View Article • Google Scholar doi: 10.1023/a:1016599411804 12211331

[20] Delamater PL, Street EJ, Leslie TF, Yang YT, Jacobsen KH. Complexity of the basic reproduction number (R0). Emerging infectious diseases. 2019 Jan;25(1):1. View Article • Google Scholar doi: 10.3201/eid2501.171901 30560777 PMC6302597

[21] Chowell G, Nishiura H. Quantifying the transmission potential of pandemic influenza. Physics of Life Reviews. 2008 Mar 1;5(1):50–77. View Article • Google Scholar

[22] New York Times Repository. COVID-19 Data at Github.com. https://github.com/nytimes/covid-19-data. [Accessed August 25, 2020]

[23] Data API. The Covid Tracking Project. https://covidtracking.com/data/api. [Accessed August 25, 2020].

[24] State of New Hampshire. 2020 COVID-19 Emergency Orders. Governor Chris Sununu. https://www.governor.nh.gov/news-and-media/covid-19-emergency-orders-2020. [Accessed August 26, 2020].

[25] State of Colorado. Public Health & Executive Orders. Colorado Department of Public Health and Environment. https://covid19.colorado.gov/public-health-executive-orders. [Accessed August 26, 2020].

[26] State of New Mexico. Public Health Orders and Executive Orders. Coronavirus Disease 2019 in New Mexico. https://cv.nmhealth.org/public-health-orders-and-executive-orders/. [Accessed August 26, 2020].

[27] State of Texas. Texas Counts COVID-19. Texas Health & Human Services. https://txdshs.maps.arcgis.com/apps/opsdashboard/index.html#/ed483ecd702b4298ab01e8b9cafc8b83. [Accessed August 26, 2020].

[28] State of Arizona. Arizona’s emergency response to the COVID-19 outbreak. Arizona Department of Health Services. https://www.azdhs.gov/preparedness/epidemiology-disease-control/infectious-disease-epidemiology/index.php#novel-coronavirus-home. [Accessed August 26, 2020].

[29] State of New York. Executive Orders from the Office of the Governor. Information on Novel Coronavirus. https://www.governor.ny.gov/executiveorders. [Accessed August 26, 2020].

[30] United States Census Bureau. QuickFacts: United States [Internet]. https://www.census.gov/quickfacts/fact/table/US/PST045219. [cited August 26, 2020].

[31] Nande A, Adlam B, Sheen J, Levy MZ, Hill AL. Dynamics of COVID-19 under social distancing measures are driven by transmission network structure. PLoS computational biology. 2021 Feb 3;17(2):e1008684. View Article • Google Scholar doi: 10.1371/journal.pcbi.1008684 33534808 PMC7886148

[32] Emeruwa UN, Ona S, Shaman JL, Turitz A, Wright JD, Gyamfi-Bannerman C, et al. Associations between built environment, neighborhood socioeconomic status, and SARS-CoV-2 infection among pregnant women in New York City. Jama. 2020 Jul 28;324(4):390–2. View Article • Google Scholar doi: 10.1001/jama.2020.11370 32556085 PMC7303894

[33] State of Texas. Texas Executive Orders and Public Health Disaster Declaration. Texas Department of State Health Services. https://dshs.texas.gov/coronavirus/execorders.aspx. [Accessed August 26, 2020].

[34] Centers for Disease Control and Prevention. Daily Updates of Totals by Week and State. National Center for Health Statistics. https://www.cdc.gov/nchs/nvss/vsrr/covid19/index.htm. [Accessed August 26, 2020].

[35] Stokes EK, Zambrano LD, Anderson KN, Marder EP, Raz KM, Felix SE, et al. Coronavirus disease 2019 case surveillance—United States, January 22–May 30, 2020. Morbidity and Mortality Weekly Report. 2020 Jun 19;69(24):759. View Article • Google Scholar doi: 10.15585/mmwr.mm6924e2 32555134 PMC7302472

[36] Centers for Disease Control and Prevention. COVID-19 Pandemic Planning Scenarios. Atlanta: CDC 24/7: Savings Lives, Protecting People. https://www.cdc.gov/coronavirus/2019-ncov/hcp/planning-scenarios.html. [Accessed July 10, 2020]

[37] Centers for Disease Control and Prevention. Social Distancing: Keep a Safe Distance to Slow the Spread. Atlanta, GA: CDC 24/7: Savings Lives, Protecting People. https://www.cdc.gov/coronavirus/2019-ncov/prevent-getting-sick/social-distancing.html. [Accessed July 15, 2020].

[38] Centers for Disease Control and Prevention. Preparing for COVID-19 in Nursing Homes. Atlanta: CDC 24/7: Saving Lives, Protecting People. https://www.cdc.gov/coronavirus/2019-ncov/hcp/long-term-care.html. [Accessed August 2, 2020].

[39] Stutt RO, Retkute R, Bradley M, Gilligan CA, Colvin J. A modelling framework to assess the likely effectiveness of facemasks in combination with ‘lock-down’in managing the COVID-19 pandemic. Proceedings of the Royal Society A. 2020 Jun 24;476(2238):20200376. View Article • Google Scholar doi: 10.1098/rspa.2020.0376 32821237 PMC7428039

[40] Allen HL, Burton W. Stop using the term ‘social distancing’–Start talking about ‘physical distancing, social connection.’. Health Affairs Blog. https://www.healthaffairs.org/do/10.1377/hblog20200424. 2020;213070. View Article

[41] Weitz JS, Dushoff J. Modeling post-death transmission of Ebola: challenges for inference and opportunities for control. Scientific reports. 2015 Mar 4;5(1):1–7. View Article • Google Scholar doi: 10.1038/srep08751 25736239 PMC4348651

[42] Loli Piccolomini E, Zama F. Monitoring Italian COVID-19 spread by a forced SEIRD model. PloS one. 2020 Aug 6;15(8):e0237417. View Article • Google Scholar doi: 10.1371/journal.pone.0237417 32760133 PMC7410324

[43] Lattanzio S, Palumbo D. Lifting restrictions with changing mobility and the importance of soft containment measures: a seird model of covid-19 dynamics. Covid Economics. 2020 May 11:1–41. View Article • Google Scholar

[44] Keeling MJ, Rohani P. Modeling infectious diseases in humans and animals. Princeton university press; 2011 Sep 19. View Article • Google Scholar

[45] Vynnycky E, White R. An introduction to infectious disease modelling. OUP oxford; 2010 May 13. View Article • Google Scholar

[46] Powell A. Five simple steps would tame COVID-19. The Harvard Gazette. 2020 August 6. View Article

[47] Fernández-Villaverde J, Jones CI. Estimating and simulating a SIRD model of COVID-19 for many countries, states, and cities. National Bureau of Economic Research; 2020 May 7. View Article • Google Scholar 10.1016/j.jedc.2022.104318 PMC8799324 35125563

[48] Bar-On YM, Flamholz A, Phillips R, Milo R. Science Forum: SARS-CoV-2 (COVID-19) by the numbers. Elife. 2020 Mar 31;9:e57309. View Article • Google Scholar doi: 10.7554/eLife.57309 32228860 PMC7224694

[49] Elflein, J. Infection rates from viruses involved in outbreak worldwide as of 2020. Statista. https://www.statista.com/statistics/1103196/worldwide-infection-rate-of-major-virus-outbreaks/. [Accessed August 24, 2020].

[50] Cano OB, Morales SC, Bendtsen C. Covid-19 modelling: the effects of social distancing. medRxiv. 2020 Jan 1. View Article • Google Scholar

[51] Arriola LR, Grossman AN. Ethnic marginalization and (non) compliance in public health emergencies. 2020. View Article • Google Scholar

[52] Treisman, R. (2020, August 5). Los Angeles Mayor Says City May Shut Off Water, Power At Houses Hosting Large Parties. National Public Radio. View Article

[53] General Accounting Office. Data Quality and Considerations for Modeling. GAO Technology Assessment. https://www.gao.gov/assets/gao-20-635sp-highlights.pdf. [Accessed July 10, 2020].

