# Supplementary figures and images for: Mitigation strategies and compliance in the COVID-19 fight; how much compliance is enough?
Source: PLoS One. 2021 Aug 9;16(8):e0239352. doi: 10.1371/journal.pone.0239352 (PMC8351990; doi:10.1371/journal.pone.0239352)

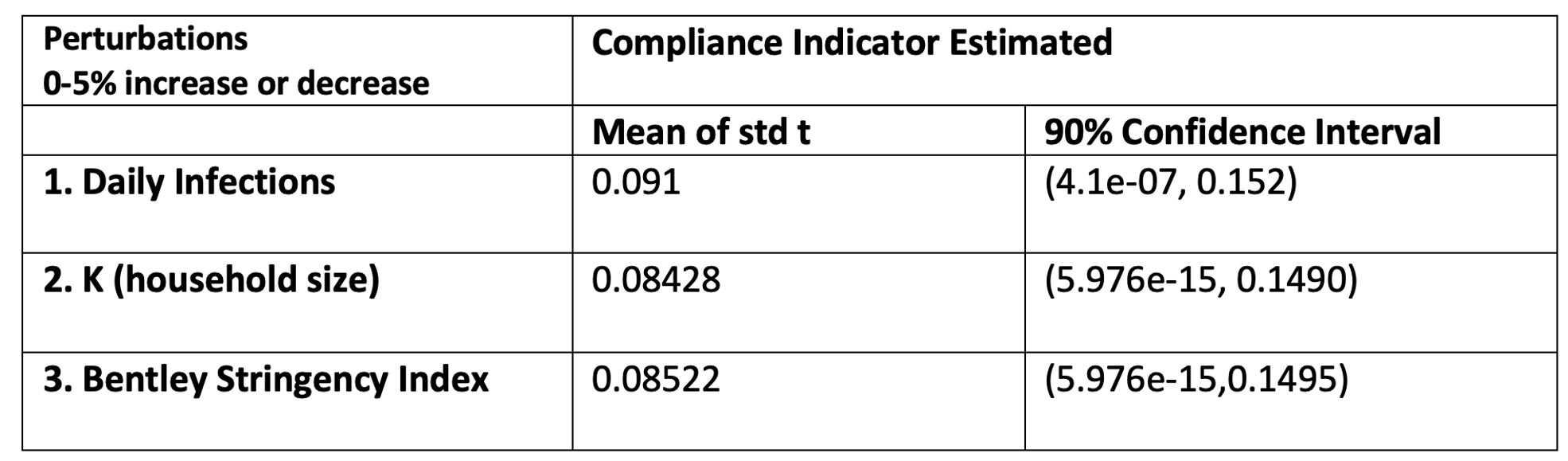

Supplement: S1 Table — Legend: This table shows the effect on the Compliance Indicator by perturbing 3 sets of numbers at a time: the daily infection, household size, k and the Bentley Stringency Index. • N refers to the number of simulations done. • std_t is the standard deviation of 1000 simulations at time t. • Mean of std_t is the average of all the 1000 standard deviations obtained. • The 90% Confidence Interval is obtained by sorting all the std_t in ascending order and then computing the 5th and 95th percentiles. (TIF) [file pone.0239352.s001.tif]

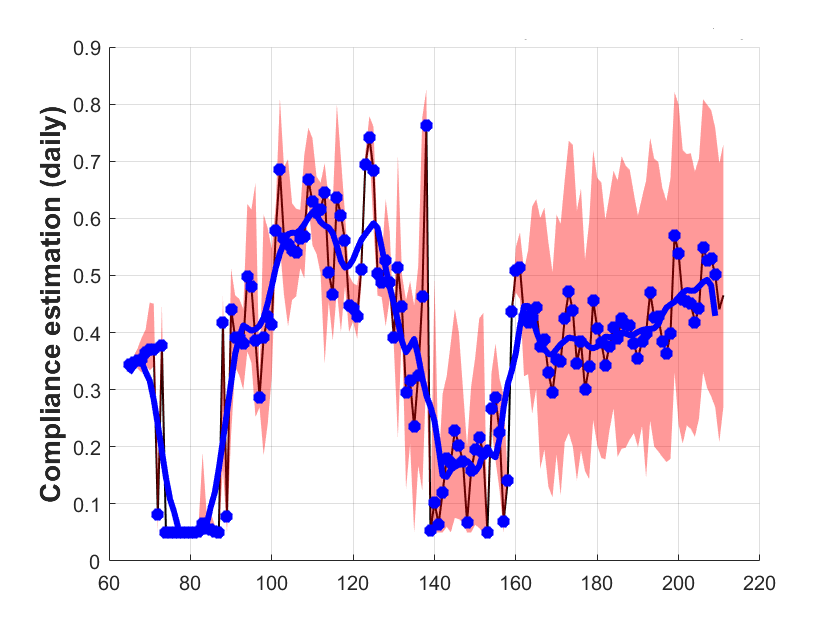

Supplement: S1 Fig — Legend: This figure shows the simulation on NY observed infection cases from Mar to July. A random inflation rate (uniformly from 0% to 5%) was applied to the observed infection cases for each day. The daily compliance rate was estimated from the algorithm. With N = 1000 simulation, the 90% confidence band for the daily compliance rate was shown in the figure, as well as the 7-day average (blue dots) and spline smoothing trend (blue line). (TIF) [file pone.0239352.s002.tif]

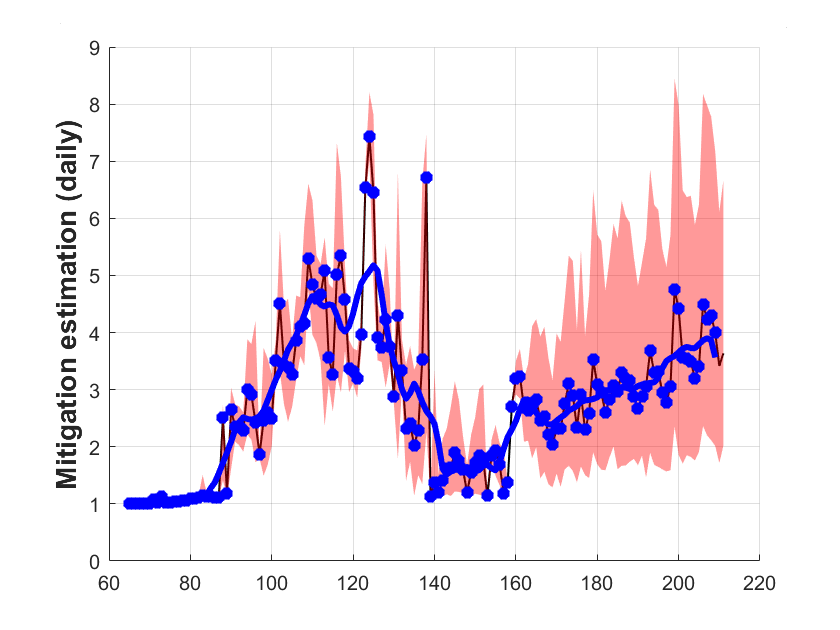

Supplement: S2 Fig — Legend: This figure shows the simulation on NY observed infection cases from Mar to July. A random inflation rate (uniformly from 0% to 5%) was applied to the observed infection cases for each day. The daily compliance rate was estimated from the algorithm. With N = 1000 simulation, the 90% confidence band for the daily mitigation function from the daily compliance rate was shown in the figure, as well as the 7-day average (blue dots) and spline smoothing trend (blue line). (TIF) [file pone.0239352.s003.tif]
