# Supplementary material for: Mitigation strategies and compliance in the COVID-19 fight; how much compliance is enough?
Source: PLoS One. 2021 Aug 9;16(8):e0239352. doi: 10.1371/journal.pone.0239352 (PMC8351990; doi:10.1371/journal.pone.0239352)
Supplement: S1 Appendix — (DOCX) [file pone.0239352.s004.docx]

**S1 Appendix**

**Appendix A**

**Table 2. Items included in the Oxford and Bentley state specific Stringency Indices.**

| **Oxford State SI Item*** | **In Oxford SI Model (Y/N)** | **In Bentley SI Model (Y/N)** | **Coding Categories** | **Consider Statewide Implementation** |
| --- | --- | --- | --- | --- |
| **C1 - School Closing Requirement** | Y | Y | 0 -No measures  1 -recommend closing  2 -Require closing(only some levels or categories, e.g. just high school, or just public schools)  3 -Require closing all levels | 0 – Targeted Areas  1- Statewide |
| **C2 - Workplace Closing Requirement** | Y | Y | 0 - No measures  1 - recommend closing (or work from home)  2 - require closing (or work from  home) for some sectors or categories of workers  3 - require closing (or work from home) all-but-essential workplaces (eg grocery stores, doctors)  No data - blank | 0 – Targeted Areas  1- Statewide |
| **C3 - Cancelling of Public Events** | Y | Y | 0- No measures  1 - Recommend cancelling 2 - Require cancelling | 0 – Targeted Areas  1- Statewide |
| **C4 - Restrictions on Gatherings to 1,000 or fewer people** | Y | Y | 0 - No restrictions  1 - Restrictions on very large gatherings (the limit is above 1000 people)  2 - Restrictions on gatherings between 100-1000 people  3 - Restrictions on gatherings between 10-100 people  4 - Restrictions on gatherings of less than 10 people | 0 – Targeted Areas  1- Statewide |
| **C5 - Closing of Public Transportation** | Y | Y | 0 - No measures  1 - Recommend closing (or significantly reduce volume/route/means of transport available)  2 - Require closing (or prohibit most citizens from using it) | 0 – Targeted Areas  1- Statewide |
| **C6 - Stay-at-Home or “Shelter-in-Place” Requirement** | Y | Y | 0 - No measures  1 - recommend not leaving house  2 - require not leaving house with exceptions for daily exercise, grocery shopping, and ‘essential’ trips  3 - Require not leaving house with minimal exceptions (e.g. allowed to leave only once every few days, or only one person can leave at a time, etc.) No data - blank | 0 – Targeted Areas  1- Statewide |
| **C7 - Restrictions on Internal Movement through Public Transportation** | Y | N | 0 - No measures  1 - Recommend closing (or significantly reduce volume/route/means of transport)  2 - Require closing (or prohibit most people from using it) | 0 – Targeted Areas  1- Statewide |
| **C8 - Internal Travel Controls through Screening or Quarantine from High Risk Regions** | Y | N | 0 - No measures  1 - Screening  2 - Quarantine arrivals from high-risk regions  3 - Ban on high-risk regions  4 - Total border closure | 0 – Targeted Areas  1- Statewide |
| **E1 - Income Support for those who lose their jobs or cannot work** | Y | N | 0 - no income support  1 - government is replacing less than 50% of lost salary (or if a flat sum, it is less than 50% median salary)  2 - government is replacing more than 50% of lost salary (or if a flat sum, it is greater than 50% median salary)    No data - blank  0 - formal sector workers only  1 - transfers to informal sector workers too No data - blank | Not Considered statewide implementation |
| **E2 - Debt/Contract Relief for Households by freezing financial obligations (utilities, loans and evictions)** | Y | N | 0 - No  1 - Narrow relief, specific to one kind of contract  2 - broad debt/contract relief | Not Considered statewide implementation |
| **H1 - Public Officials Commenting or Coordinating Public Information Campaigns about COVID-19** | Y | N | 0 -No COVID-19 public information campaign  1 - public officials urging caution about COVID-19  2 - coordinated public information campaign (e.g. across traditional and social media) | 0 – Targeted Areas  1- Statewide |
| **H2 - Communicating policy on COVID-19 Testing** | Y | Y | 0 – No testing policy  1 – Only those who both (a) have symptoms AND (b) meet specific criteria (eg key workers, admitted to hospital, came into contact with a known case, returned from overseas)  2 – testing of anyone showing COVID-19 symptoms  3 – open public testing (eg “drive through” testing available to asymptomatic people) | 0 – Targeted Areas  1- Statewide |
| **H3 - Implementing Contact Tracing** | Y | Y | 0 - No contact tracing  1 - Limited contact tracing - not done for all cases  2 - Comprehensive contact tracing - done for all cases | Not Considered statewide implementation |
| **New Items Added to Current Study** | | | |  |
| **C9 - State Border Restrictions Requiring Self-Quarantine** | N | Y | 0 - No Measure  1- Recommend Individuals arriving from other states to self-quarantine  2-Require individuals arriving from other states to self-quarantine | 0 – Targeted Areas  1- Statewide |
| **C10 - Limiting Nursing Home Visitation** | N | Y | 0 - No Measure  1 - Recommend limit nursing home visitation  2 - Require limit nursing home visitation | 0 – Targeted Areas  1- Statewide |
| **C11 - Social Distancing Practice of Six Feet or Farther Apart** | N | Y | 0 - No Measure  1 - Recommend limiting to six feet distance in public  2 - Require limiting to six feet distance in public | 0 – Targeted Areas  1- Statewide |
| **H6 - Face Mask Worn in Public Requirement** | N | Y | 0 - No Mention of face masks  1 - Recommend wearing face mask in public  2 - Require wearing face mask in public | 0 – Targeted Areas  1- Statewide |

*C refers to containment or closure strategies, E refers to economic response policies and H refers to health systems strategies. Targeted refers to specific areas of the state the policy applies, while general indicates the policy is implemented statewide

**Appendix B**

**Creation of new items in the Bentley modification of the Oxford Stringency Index.**

The Bentley Stringency Index (BSI) is compiled from 12 items whereas the Oxford state Stringency Index (OSI) is based on 13 items [14]. We modified the Oxford state Stringency Index by dropping five items and adding four. We dropped item C7 (restriction on internal movement through public transportation) because this policy was already included in item C5 (closing of public transportation). We also dropped C8 (internal travel controls through screening or quarantine from high risk regions) because we were not aware of states closing borders entirely or banning the entry of individuals from high risk regions. Even Rhode Island’s attempt to target cars with New York state license plates in order to impose a 14 day quarantine on visitors from New York was rescinded [29]. We also set aside items E1 (income support for those who lose their jobs), E2 (debt/contract relief for households) and H1 (public official commenting or coordinating public information campaigns about Covid-19) because we felt they did not pertain to containment or closure. We added a total of four items to the Stringency Index. These items pertain to states imposing a mandatory self-quarantine on visitors from other states (C9), limiting nursing home visitation (C10), recommending a social distancing practice in public of at least six feet (C11) and policy on face mask covers (H6).

The daily Stringency Index score is tabulated as an average of 12 sub-indices drawn from the containment and closure and health systems items in the Oxford inventory scaled from 0 to 100. Additional weight is given to 10 of the 12 policy items that could potentially be implemented statewide. Because the four items we created (see Appendix A Table) resulted in two more items than the original Oxford SI that could be implemented statewide, we modified the weight on which the final Stringency Index score is based. This is done by weighing the Likert scale points of the 10 indicators that are designated for statewide or targeted implementation using the formula outlined by the Oxford group.

$$W_{r}=\frac{1}{10}\sum_{j=1}^{10} \frac{1}{\left( 2.95+1 \right)}\approx0.30$$

Our weight of 0.30 was very close to the ones calculated by Oxford (0.29), based on eight items, for use in the original index. The weights are then employed to create sub-indices of those items scaled to 100 in the same manner indicated from Oxford.


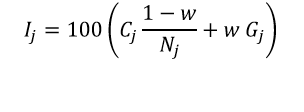


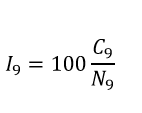
For the two items without statewide implementation as an option, sub-indices were calculated simply as a scaled function of 100 multiplied by the ratio of the raw scores to their maximum point value.

The final Stringency Index score is then an average of the all sub-indices.
